# Tranexamic acid for the prevention of postpartum bleeding in women with anaemia:  Statistical analysis plan for the WOMAN-2 trial: an international, randomised, placebo-controlled trial

**DOI:** 10.12688/gatesopenres.14529.1

**Published:** 2023-05-12

**Authors:** Tim Collier, Haleema Shakur-Still, Ian Roberts, Eni Balogun, Oladapo Olayemi, Folasade Adenike Bello, Rizwana Chaudhri, Projestine Muganyizi, WOMAN-2 Trial Collaborators

**Affiliations:** 1 CTU Global Health Trials Group, London School of Hygiene & Tropical Medicine, London, WC1E 7HT, UK; 2 College of Medicine, University of Ibadan, Ibadan, 200212, Nigeria; 3 Global Institute of Human Development, Shifa Tameer-e-Millat University, Islamabad, Pakistan; 4 Department of Obstetrics and Gynaecology, Muhimbili University of Health and Allied Sciences, Dar es Salaam, Tanzania

**Keywords:** antifibrinolytic, clinical trial, anaemia, postpartum haemorrhage, tranexamic acid

## Abstract

**Background: **Postpartum haemorrhage (PPH) is responsible for over 50,000 maternal deaths every year. Most of these deaths are in low- and middle-income countries. Tranexamic acid (TXA) reduces bleeding by inhibiting the enzymatic breakdown of fibrin blood clots. TXA decreases surgical bleeding and reduces deaths from bleeding after traumatic injury. When given within three hours of birth, TXA reduces deaths from bleeding in women with PPH. However, for many women, treatment of PPH is too late to prevent death. World-wide, over one-third of pregnant women are anaemic and many are severely anaemic. These women have an increased risk of PPH and are more likely to die if PPH occurs. There is an urgent need to identify ways to prevent severe postpartum bleeding in anaemic women. The WOMAN-2 trial will quantify the effects of TXA on postpartum bleeding in women with anaemia.

**Results: **This statistical analysis plan (version 1.0; dated 22 February 2023) has been written based on information in the WOMAN-2 Trial protocol version 2.0, dated 30 June 2022. The primary outcome of the WOMAN-2 trial is the proportion of women with a clinical diagnosis of primary PPH. Secondary outcomes are maternal blood loss and its consequences (estimated blood loss, haemoglobin, haemodynamic instability, blood transfusion, signs of shock, use of interventions to control bleeding); maternal health and wellbeing (fatigue, headache, dizziness, palpitations, breathlessness, exercise tolerance, ability to care for her baby, health related quality of life, breastfeeding); and other health outcomes (deaths, vascular occlusive events, organ dysfunction, sepsis, side effects, time spent in higher level facility, length of hospital stay, and status of the baby).

**Conclusions: **WOMAN-2 will provide reliable evidence about the effects of TXA in women with anaemia.

**Registration: **WOMAN-2 was prospectively registered at the International Standard Randomised Controlled Trials registry ( ISRCTN62396133) on 07/12/2017 and ClinicalTrials.gov on 23/03/2018 ( NCT03475342).

## Introduction

World-wide, about half a billion women of reproductive age are anaemic and 20 million are severely anaemic ^1^. The prevalence is highest in South Asia and Sub-Saharan Africa where approximately half of all pregnant women are anaemic ^2^. Anaemia is a strong risk factor for postpartum haemorrhage (PPH) (often defined as blood loss ≥500 ml in the 24 hours after birth), the leading cause of maternal death worldwide ^3^. Global efforts to reduce the prevalence of anaemia have not been successful and in most countries the prevalence of anaemia in women of reproductive age has increased ^4^. More effective methods to reduce the prevalence of anaemia are urgently needed but we also need interventions to reduce the risk of postpartum haemorrhage in anaemic women.

Tranexamic acid (TXA) reduces bleeding by inhibiting the breakdown of blood clots. When given within three hours of birth, TXA reduces deaths from bleeding in women with PPH ^5^. Expanding the use of TXA to women without established PPH could have major benefits but we must also consider potential risks. Due to the increased tendency of their blood to clot and the pressure on pelvic veins from the expanding uterus, pregnant women have an increased risk of venous thrombosis ^6^. When considering the use of TXA in women without PPH, we must carefully consider the balance of risks and benefits. Because anaemia decreases the ability of the blood to clot and increases the risk of bleeding, balance of benefits and risks should be more favourable in women with anaemia. The WOMAN-2 trial will quantify the effects of TXA on postpartum bleeding and thrombosis in women with anaemia ^7^.

## Protocol

### Ethical approval and registration

The WOMAN-2 Trial was approved by the London School of Hygiene and Tropical Medicine Research Ethics Committee (REF: 15194) on 10 May 2018; by the National Health Research Ethics Committee of Nigeria (NHREC/01/01/2007-29/09/2019) on 29 September 2019; by the National Bioethics Committee of Pakistan on 27 November 2018 (NBC-340); by the National Institute of Medical Research of Tanzania (NIMR/HQ/R.8a/Vol.IX/3767) on 19 August 2021; by the University of Zambia Biomedical Research Ethics Committee (REF. 001-04-19) on 02 February 2019. The trial was prospectively registered at the International Standard Randomised Controlled Trials registry (ISRCTN62396133) on 07 December 2017 and ClinicalTrials.gov on 23 March 2018 (NCT03475342).

### Trial design and patients

The WOMAN-2 trial is a randomised, placebo-controlled superiority trial of the effects of TXA in women with moderate or severe anaemia who are giving birth vaginally ^7^. Women with moderate or severe anaemia giving birth in participating hospitals will be randomly allocated to receive 1g of TXA or matching placebo (sodium chloride 0.9%) by intravenous injection as soon as possible (and no later than 15 minutes) after the umbilical cord is cut or clamped. Women from hospitals where maternal anaemia is common, mostly South Asia and Africa, will be enrolled. All participating hospitals have the facilities to provide comprehensive essential obstetric care as defined by the World Health Organization.

### Randomisation and masking

The randomisation codes were prepared by an independent statistician who was not involved in the conduct of the trial. The codes were sent to the trial drug manufacturer so that treatment packs could be prepared in accordance with the randomisation list. The codes were also sent to the Sponsor’s representative, also not associated with the trial, as a manual back-up for emergency unblinding. The trial staff (coordinating centres and sites) did not have access to the randomisation codes until after final database lock. In those rare instances where unblinding was requested, a member of the trial team was made aware of whether that particular participant received TXA or placebo so that they could communicate this information to the health professional requesting the unblinding.

The TXA for the trial was purchased on the open market. TXA has a marketing authorisation which guarantees that drug manufacture and release comply with Good Manufacturing Practice (GMP). A GMP certified manufacturer prepared the matching placebo (sodium chloride 0.9%). Tranexamic acid and placebo ampoules and packaging were identical. A clinical trial supplies company conducted the blinding and first-stage Qualified Person release. The blinding process involved replacing the manufacturer’s label with the clinical trial label. Other than the randomisation number (used for pack identification) the label text was identical on all ampoules. To check the blinding, known tranexamic acid was compared with blinded samples from a random set of treatment packs to determine which were tranexamic acid. The samples were then un-blinded to confirm accuracy of the labelling.

### Trial procedures

**Figure 1.**
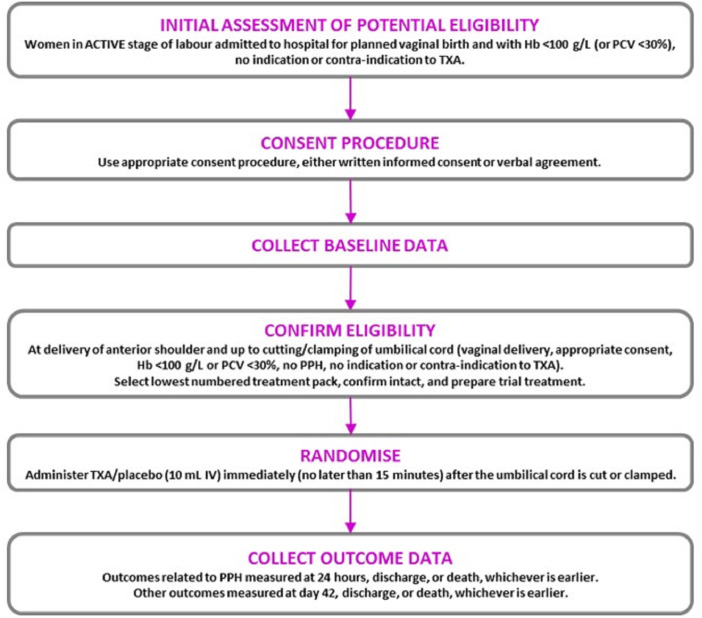
WOMAN-2 Trial Overview.

Women who were eligible for inclusion were randomly allocated to receive tranexamic acid or placebo (sodium chloride 0.9%) by slow intravenous injection. Once eligibility was confirmed (after the birth of the baby’s anterior shoulder and up to when the cord is cut), the next lowest consecutively numbered pack was taken from a box of 20 treatment packs. The participant was considered randomised when the administration of the treatment started. Each site kept a log of women enrolled into the trial. Site investigators were asked to explain any out-of-sequence treatment pack use. An overview of the trial is shown in Figure 1.

### Trial overview

***Sample size*:** To estimate the sample size, we assumed a risk of PPH in the placebo group of about 9%. We estimated that a trial with around 15,000 women would have 85% power to detect a 15% relative risk reduction in PPH (risk ratio 0.85) using a two-side alpha of 5%. We had initially estimated a sample size of 10,000 women on the basis of a 25% relative risk reduction in PPH (risk ratio 0.75) and a risk of PPH in the placebo group of 10% based on the available literature ^8^. However, whilst the WOMAN-2 trial was in-progress, several trials of TXA for the prevention of PPH were published that each included over 1,000 women ^9– 11^. When we pooled the results of these trials, we found that the treatment effect, although still clinically important, may be more modest than we had first estimated. The larger trials showed that it would be more reasonable to expect a 15% relative reduction in risk of PPH with TXA. Nevertheless, if such a simple and widely practicable treatment reduced the risk of PPH by 15% this would have major health implications, preventing millions of PPH cases each year world-wide. We also had more information about the event rate. Assuming that TXA reduces the risk of PPH by 15% and using the overall event rate (i.e., the pooled treatment and placebo event rate) we estimated the placebo group event rate in the trial to be around 9%. Because PPH is such an important maternal health problem for which TXA would offer an inexpensive and widely practicable solution, we wanted to avoid the possibility of missing a real but more modest treatment benefit. The sample size re-estimation was carried out fully blinded to any interim results.

## Statistical analysis plan

This statistical analysis plan (version 1.0; dated 22 February 2023) has been written based on information contained in the WOMAN-2 Trial protocol version 2.0, dated 30 June 2022.

### Trial profile

We will report the flow of study participants through the trial in a Consolidated Standards of Reporting Trials (CONSORT) diagram. We will start with the total number of women randomised into the trial by treatment arm. Within each treatment arm we will detail the number of women who withdraw consent, the number for whom baseline data were collected, the number lost to follow up, and the number of women for whom outcome data were collected. We will report the number of women included in the primary and secondary analyses, and the reasons for any post-randomisation exclusions. We will count women that did not fulfil the eligibility criteria or did not receive their allocated treatment as having deviated from the protocol, but their data will be included in the intention to treat analysis. If a woman or her representative withdraws consent for data collection, we will use only data up to the point of withdrawal in the analysis.

### Baseline characteristics

Baseline patient characteristics will be summarised by randomised group. Categorical variables will be summarised using frequencies and percentages. Quantitative variables will be summarised using the number of observations, mean and standard deviation, median and interquartile range, and range. The number of missing values will be reported.

### Primary outcome

The primary outcome for WOMAN-2 is a clinical diagnosis of primary PPH ^12^. This is defined as an estimated blood loss of more than 500 mL or any blood loss sufficient to compromise haemodynamic stability within 24 hours of administration of the trial medication. Haemodynamic instability is based on clinical judgement and assessed using clinical signs (low systolic blood pressure, tachycardia, reduced urine output). The cause of PPH will be described.

### Primary analysis

The primary analysis will compare the proportion of women with PPH in those allocated tranexamic acid versus those allocated placebo, on an intention to treat basis (irrespective of whether or not they received the allocated treatment). The number and percentage of women with a PPH will be reported by treatment group. Percentages will be calculated from those with available data on the primary endpoint. We will report the number of women with missing data. The primary analysis results will be presented as a relative risk with a 95% confidence interval (CI) calculated using a binomial regression model with a log link, including randomised treatment as the only covariate. Since we will be judging the success of the trial on the primary endpoint alone there will be no adjustment for multiplicity. The primary cause of PPH is recorded based on the judgment of the responsible clinician. We will also report the effect of TXA on cause specific PPH (atony, placenta implantation abnormalities, tears, retained placental tissue, uterine rupture, other and unknown). However, the primary analysis will be for all-cause PPH. 

***Sensitivity analysis*:** Two sensitivity analyses will be carried out for the primary endpoint.

(i) The analysis of the primary outcome will be repeated after adjusting for important baseline risk factors for PPH as a sensitivity analysis. The following baseline variables will be included as covariates in the binomial regression model along with randomised treatment: age, pre-birth haemoglobin, previous PPH, number of babies (in this pregnancy), placental abnormality, and any birth canal trauma.

(ii) Although women with PPH prior to randomization were not eligible (since this bleeding could not be affected by the trial treatment), some women had on-going antepartum bleeding. Because it can be difficult to distinguish between on-going ante-partum bleeding and PPH prior to randomisation, we will repeat the analysis excluding women who had a continuing (ante-partum) haemorrhage. 

### Missing data

Because we expect loss to follow-up to be minimal (<0.5% for the primary outcome), we will not impute missing values for the primary outcome.

### Subgroup analyses

A limited number of prespecified subgroup analyses will be performed for the primary outcome only. We will report heterogeneity p-values for all subgroup analyses regardless of statistical significance. This approach to subgroup analyses is designed to alleviate concerns about multiplicity.

We will carry out the following subgroup analyses: i) severity of anaemia (moderate versus severe), ii) antepartum bleeding (any versus none), iii) type of birth (traumatic versus non-traumatic), iv) baseline risk of PPH (low, intermediate, high), v) use of pain control (any versus none). The biological rationale for the sub-group analyses is described in detail below. Unless there is strong evidence against the null hypothesis of homogeneity of effects (i.e., p<0.001) the overall effect estimate will be considered the most reliable guide to the approximate effect in each of the subgroups.

i) Severity of anaemia (moderate versus severe): There are plausible biological reasons why anaemia might modify the effect of TXA on the risk of post-partum bleeding. In vitro studies show that anaemic blood clots are more prone to fibrinolysis. Fibrin clots formed in the presence of red blood cells have a tight conformation made of thin fibrin strands which are resistant to fibrinolysis. Clots formed in the absence of red cells are made of thicker fibrin strands in a looser conformation that is more rapidly broken down by plasmin ^13, 14^. Red blood cells also appear to shield fibrin strands from lysis by plasmin, a process known as steric hindrance ^14^. Animal studies comparing the effects of TXA on bleeding in anaemic and non-anaemic mice show that TXA is more effective in the presence of anaemia ^15^. Cohort studies in pregnant women recruited in the last trimester of pregnancy and followed until after childbirth support these laboratory observations ^16^. Compared to women with a Hb >100 g/L, the D-dimer concentration (the product of fibrinolysis) was 8% higher in women with moderate anaemia (70–100 g/L) and 27% higher in those with severe anaemia (<70 g/L). If fibrinolysis is worse in women with anaemia, we might expect a greater effect of TXA in anaemic women. In the light of these results, we conducted further *in-vitro* studies to examine the possibility that anaemia could modify the effect of TXA. These did not show that TXA was more effective in the presence of anaemia. On the contrary, they suggested that RBCs might increase the anti-fibrinolytic potency of TXA ^17^. Furthermore, the POISE-3 trial of tranexamic acid in non-orthopaedic surgery found a large reduction in the risk of major bleeding with TXA. A pre-specified subgroup analysis according to pre-operative haemoglobin level (<120 g/L versus >120 g/L) did not show strong evidence of heterogeneity. Indeed, the treatment benefit was greater in non-anaemic patients (RR = 0.65) than in anaemic patients (RR = 0.83) ^18^. In the Woman-2 trial, we will examine the hypothesis that anaemia modifies the effect of TXA in a pre-specified subgroup analysis (moderate versus severe anaemia). However, given the recent results, we do not expect substantial heterogeneity.

ii) Antepartum bleeding (any versus none): There is strong evidence that the effect of TXA in acute severe bleeding varies by time to treatment, with treatment started soon after bleeding onset being more effective ^19^. Although most women with severe postpartum bleeding start bleeding after the birth, in some women bleeding starts before birth (e.g., placental abruption or placenta previa) and continues after the birth. In these women, post-partum bleeding is a continuation of ante-partum bleeding. If time from bleeding onset modifies the effect of TXA, we might expect that TXA would be less effective in women with ante-partum haemorrhage. We will examine this hypothesis by conducting an analysis of the effects of TXA on the risk of PPH according to whether there was evidence of antepartum bleeding or no antepartum bleeding.

iii) Type of birth (traumatic versus non traumatic): Tranexamic acid reduces surgical bleeding and reduces deaths from bleeding in trauma, whether from accidents or violence. This raises the possibility that the benefits of TXA in PPH primarily reflect the traumatic nature of childbirth. Maternal injury may arise during the passage of the infant through the birth canal or due to actions taken by health care workers (e.g., episiotomy). We will examine this hypothesis by conducting an analysis of the effects of TXA on the risk of PPH according to whether the birth was traumatic or not. For this analysis, traumatic birth is defined as perineal, vaginal or cervical tearing, uterine rupture or episiotomy.

iv) Baseline risk of PPH (low, intermediate, high): Knowing if the effect of TXA on the risk of PPH varies according to whether a woman is at low, medium or high risk would help clinicians to decide who (if any) should receive this treatment. To explore this, we will follow the approach used for previous analyses of how the effects of TXA varies by baseline risk in bleeding patients. We will develop a prognostic model to estimate the baseline risks of postpartum haemorrhage after childbirth using the data from the Woman-2 trial. We will only use baseline characteristics collected before randomisation as potential predictors and will use data from both the treatment and placebo groups to improve precision. We will use the backward stepwise method and remove one at a time, variables for which there is no evidence of association (P-value for the Wald test >0.05). We will assess the performance of the model by estimating discrimination and calibration. Using the model, each woman will be assigned to a category of risk (low, intermediate, or high) depending on the distribution of the outcomes. We will calculate effect estimates within each category which we will examine for statistical evidence of homogeneity. Previous analyses of the effects of TXA in severely bleeding patients, suggests that the effects of TXA does not vary by baseline risk ^20^. Based on this evidence, we do not anticipate statistical heterogeneity in the effects of TXA by the baseline risks of PPH.

v) Use of pain control (any versus none): Childbirth is intensely painful. Pain and fear in labour can provoke a stress response with maternal adrenaline levels up to six times higher during labour ^21, 22^. Adrenaline in turn can activate fibrinolysis. Tissue plasminogen activator (t-PA), released from the endothelium in response to adrenaline, converts plasminogen into the active fibrinolytic enzyme plasmin. Chandler gave adrenaline injections to healthy volunteers and found that t-PA levels rise linearly with increasing adrenaline ^23^. The association between adrenaline concentrations and fibrinolysis is also seen in trauma ^24, 25^. High adrenaline concentrations are associated with increased fibrinolysis and higher mortality ^24^. Effective pain control in labour can reduce the maternal adrenaline response. There is a significant drop in plasma adrenaline after an epidural block compared to pre-block values ^26^. In one study of labouring women, before epidural block, the mean (SEM) plasma adrenaline level was 280 ± 49 pg/mL, a level similar to that observed in moderately injured trauma patients ^27^. After the block, maternal adrenaline values fell by 56%. The use or non-use of pain control in labour might therefore affect the degree of fibrinolysis after childbirth. We hypothesize that tranexamic acid will be most effective in women giving birth without pain control and will examine this in a subgroup analysis.

### Key secondary outcomes

The following four key secondary outcomes will be assessed at 24 hours after administration of trial treatment or discharge from hospital, whichever is earlier. 

(i) Postpartum blood loss (clinical estimation of blood loss since administration of trial treatment)

(ii) Haemoglobin (corrected for blood transfusion). 

(iii) Any vascular occlusive events: pulmonary embolism (PE), deep vein thrombosis (DVT), stroke and myocardial infarction (MI).

 (iv) Death or Near miss (as defined by the World Health Organization (WHO)).

### Analysis of key secondary outcomes

(i) Postpartum blood loss: Two approaches will be used to compare estimated postpartum blood loss between the randomised groups. Firstly, we will estimate the difference in mean blood loss and a 95% CI using a two-sample t-test. Secondly, we will categorise blood loss into five categories (0–249, 250–499, 500–999, 1000–1499 and 1500+ ml). We will report the number and percentage of women in each category by randomised group. We will estimate an odds ratio and 95% CI using an ordinal logistic regression model. We will show graphically the proportion of patients in each category by randomised treatment using a stacked bar chart.

(ii) Haemoglobin: Post-partum haemoglobin will be corrected for the effect of blood transfusion using the method described by Roubinian ^28^. Two approaches will be used to compare the corrected haemoglobin between the randomised groups. Firstly, we will use analysis of covariance to estimate the difference and 95% CI in the mean change in haemoglobin adjusting for baseline haemoglobin. Secondly, we will categorise the corrected haemoglobin into four categories of anaemia (none = Hb≥110, mild = Hb 100–109, moderate = Hb 70–99, Severe = Hb <70) according to WHO guidelines ^29^. We will report the number and percentage of women in each category by randomised group. We will estimate an odds ratio and 95% CI using an ordinal logistic regression model. We will graphically present the proportion of patients in each category by randomised treatment using a stacked bar chart.

(iii) Any vascular occlusive event (VOE): We will report the number and percentage of women with a VOE by treatment group. We will report the number and percentage of women with any vascular occlusive event but also each specific VOE (DVT, PE, MI, Stroke) separately. A relative risk and 95% CI will be calculated using a binomial regression model with a log link, including randomised treatment as the only covariate.

(iv) Death or near-miss death related to bleeding: We will report the number and percentage of women with a death or near-miss death related to bleeding by treatment group. A relative risk and 95% CI will be calculated using a binomial regression model with a log link, including randomised treatment as the only covariate. The definition of near-miss death related to bleeding used for this analysis is:

Death or near-miss death from PPH within two days
Death from any cause
Near-miss death from PPH:
  • Severe PPH (blood loss of 1000ml+)
  • Surgical intervention for bleeding: hysterectomy for bleeding, laparotomy, embolization, uterine compression sutures, arterial ligation.
  • Failure to form clots
  • Transfusion of >5 units
  • Cardiovascular dysfunction: shock, cardiac arrest, continuous vasoactive drugs, severe hypoperfusion, severe acidosis, CPR.
  • Renal dysfunction diagnosed: oliguria non-responsive to fluids or diuretics, dialysis for acute renal failure, severe acute azotemia

### Other secondary outcomes

The following secondary outcomes will be assessed 24 hours after administration of the trial treatment or at discharge from hospital, whichever is earlier.

- Haemodynamic instability (within 24 hours of administration of trial medication): presence of haemodynamic instability based on clinical signs e.g., low blood pressure, tachycardia, reduced urine output requiring intervention (e.g., intravenous fluid).
- Shock index = Heart rate/systolic blood pressure: We will use the lowest recorded systolic blood pressure and the corresponding heart rate.

The following outcomes will be assessed at death, discharge from hospital or 42 days, whichever is earlier.

- Quality of life: the following parameters will be measured by questionnaire, overall wellbeing, ability to care for herself and her baby, and breastfeeding.
- Symptoms of anaemia: the following parameters will be assessed by questionnaire, fatigue, headache, dizziness, palpitations, breathlessness, flaring of the alae nasi.
- Expected side effects of trial medication: the following side effects will be recorded, nausea, vomiting, diarrhoea.
- Exercise tolerance: This will be assessed using the six-minute walk test.
- Blood transfusion: number of units given (units started before administration of trial medication will not be included). Information on type of transfusion will be collected.
- Use of interventions to control primary postpartum haemorrhage (medical and surgical): including uterotonics, removal of placenta/placenta fragments, intrauterine balloon tamponade, bimanual uterine compression, external aortic compression, non-pneumatic anti-shock garments, uterine artery embolization, uterine compression suture, hysterectomy and laparotomy to control bleeding.
- Organ dysfunction:
  - Cardiovascular dysfunction - shock, cardiac arrest (absence of pulse/heart beat and loss of consciousness), use of continuous vasoactive drugs, cardiopulmonary resuscitation, severe hypoperfusion (lactate >5 mmol/L or >45 mg/dL), severe acidosis (pH <7.1).
  - Respiratory dysfunction - acute cyanosis, gasping, severe tachypnea (respiratory rate >40 breaths per minute), severe bradypnea (respiratory rate <6 breaths per minute), intubation and ventilation not related to anaesthesia, severe hypoxemia (O2 saturation <90% for ≥60 min or PAO2/FiO2 <200).
  - Renal dysfunction - Oliguria non-responsive to fluids or diuretics, dialysis for acute renal failure, severe acute azotemia (creatinine ≥300 μmol/mL or ≥3.5 mg/dL).
  - Coagulation/ haematologic dysfunction - Failure to form clots, massive transfusion of blood or red cells (≥5 units), severe acute thrombocytopenia (<50,000 platelets/mL).
  - Hepatic dysfunction - jaundice in the presence of eclampsia, severe acute hyper-bilirubinemia (bilirubin >100 μmol/L or >6.0 mg/dL).
  - Neurologic dysfunction - prolonged unconsciousness (lasting ≥12 hours)/coma (including metabolic coma), stroke, uncontrollable fits/status epilepticus, total paralysis.
- Sepsis: diagnosis is based on the presence of both infection and a systemic inflammatory response syndrome (SIRS). SIRS requires two or more of the following: a) temperature <36°C or >38°C (b) heart rate >90 beats/min (c) respiratory rate >20 breaths/min (d) white blood cell count <4 x 109/L (<4000/mm³) or >12 x 109/L (>12,000/mm³)
- In-hospital death: cause and time of death will be described.
- Length of hospital stay.
- Admission to and time spent in higher level facility: higher level facilities include High Dependency and Intensive Care Units.
- Status of baby/ies: the status (dead/alive).
- Any thromboembolic events in breastfed babies (may include any venous or arterial thrombosis (thrombosis of limb artery/deep veins, renal artery/veins, pulmonary embolism, hepatic veins, caval veins, intracardiac thrombosis, portal vein, mesenteric veins/artery, cerebral veins, retinal vein, ischemic stroke, arteries, aorta, myocardial infarction, microvascular thrombosis from purpura fulminans or disseminated intravascular coagulation).

### Analysis of other secondary outcomes

The following statistical approaches will be used for the analysis of other secondary outcomes. Binary outcomes will be analysed using the same approach used for the primary outcome. The frequency and percentage of women with the outcome will be reported along with risk ratios and 95% CIs from a binomial regression model with randomised treatment as the only covariate. For continuous outcomes we will report the number of observations, mean and standard deviation by treatment group. Linear regression models (ANCOVA) with the baseline values included as a covariate (where appropriate) along with randomised treatment will be used to estimate treatment effects which will be reported as difference in means with 95% CIs. If a continuous outcome is found to violate the assumption of normality, an appropriate transformation may be applied or a non-parametric method of analysis may be used. For time to event outcomes e.g., time to death, we will report the number of events along with the estimated proportion using the Kaplan-Meier method. A Cox proportional hazards model with randomised treatment as the only covariate will be used to estimate the hazard ratio along with a 95% CI. For ordered categorical outcomes, we will report the number and percentage of women in each category. We will estimate an odds ratio and 95% CI using an ordinal logistic regression model with randomised treatment as the only covariate.

**Safety outcomes:** The number of AEs, SAEs, and SUSARs grouped by MedDRA® codes and the number of patients with at least one event will be compared between arms using a Chi-squared test (or Fisher’s exact test), with RRs and 95 % CI when these are computable.

### Analyses to be reported in separate publications

**Cost effectiveness analysis:** We will develop a decision model to assess the cost-effectiveness of tranexamic acid for the prevention of post-partum haemorrhage in women with moderate and severe anaemia. We will use data from the WOMAN-2 trial to inform model parameters, supplemented by estimates from the literature. We will estimate costs (calculated in US$), life-years, and quality-adjusted life-years (QALYs) with and without tranexamic acid, calculate incremental cost-effectiveness ratios (ICERs), and compared these to threshold values in the participating countries. Costs will be assessed from a health-care provider perspective and discounted at 3% per year in the base case analysis. We will conduct a series of one-way sensitivity analyses and probabilistic sensitivity analysis to assess the robustness of the results to parameter uncertainty.

**Individual patient data meta-analysis:** If the Woman-2 trial shows that tranexamic acid safely reduces the risk of postpartum haemorrhage in women with moderate and severe anaemia, it will be important to consider the extent to which these results can be generalised to women with mild anaemia and women who are not anaemic, who may or may not have other risk factors for PPH. To generalise results, we must consider the likely mechanism by which the treatment affected the outcome and the factors that might be relevant to this mechanism. We must also consider how the balance of benefits (e.g., bleeding risk) and potential harms (e.g., thrombotic risk) might vary according to the baseline risks of these outcomes and according to baseline maternal haemoglobin. We will explore these issues by conducting a systematic review and IPD meta-analysis that will assess the effects of TXA on the risk of life-threatening postpartum bleeding, thromboembolic events, and on other outcomes that matter to women. We will examine how the effects of TXA on life-threatening bleeding and thromboembolic events vary by the baseline risks of these outcomes, by baseline level of maternal haemoglobin, type of birth (vaginal birth versus caesarean section), and timing of treatment. A protocol and statistical analysis plan for this analysis has been presented elsewhere ^30^.

**Hierarchical endpoint analysis using win ratio:** Although maternal death is undoubtedly one of the most important consequences of severe post-partum bleeding, even very large trials will have limited power to detect the impact of the trial treatment on this critical patient outcome. Including death as a component of a composite outcome is an alternative approach but the effect of the intervention on the individual outcomes is lost and not all of the components of the composite outcome may have the same importance for the patient. Pocock and colleagues have described an analytical approach (hierarchical endpoint analysis using the win ratio) that considers endpoints in a hierarchical way thus gaining statistical power whilst respecting the relative priorities of the component outcomes ^31^. In a separate paper, we will report an analysis of the Woman-2 trial using the win ratio approach with death (1), near-miss death related to bleeding (2), hysterectomy (3) and a clinical diagnosis of PPH (4) as the ranked outcomes. 

## Data monitoring and interim analyses

An independent Data Monitoring Committee (DMC) is responsible for reviewing the progress of the WOMAN-2 trial, including recruitment, data quality, and main study outcomes and safety data. The DMC has the responsibility for deciding whether, while randomisation is in progress, the un-blinded results (or the un-blinded results for a particular subgroup) should be revealed to the TSC. The DMC Charter states that they will do this if, and only if, two conditions are satisfied: (1) the results provide proof beyond reasonable doubt that treatment is on balance either definitely harmful or definitely favourable for all, or for a particular category of, participants in terms of the major outcome; (2) the results, if revealed, would be expected to substantially change the prescribing patterns of clinicians who are already familiar with any other trial results that exist. Exact criteria for ‘proof beyond reasonable doubt’ are not, and cannot be, specified by a purely mathematical stopping rule, but they are strongly influenced by such rules. The DMC Charter is in agreement with the Peto-Haybittle ^32, 33^ stopping rule whereby an interim analysis of major endpoint would generally need to involve a difference between treatment and control of at least three standard errors to justify premature disclosure. An interim subgroup analysis would, of course, have to be even more extreme to justify disclosure. This rule has the advantage that the exact number and timing of interim analyses need not be pre-specified. In summary, the stopping rules require extreme differences to justify premature disclosure and involve an appropriate combination of mathematical stopping rules and scientific judgment.

### Data management and analysis software

All trial data is managed in accord with the WOMAN-2 trial Data Management Plan (DMP) (version 1.0) and stored in the Trial Master File. The DMP standard operating procedures conform with the London School of Hygiene & Tropical Medicine (LSHTM) policies and procedures, the Clinical Trials Unit working procedures and regulatory requirements. The clinical database management system for the WOMAN-2 trial was built to comply with ICH-GCP and uses MySQL and its accompanying manuals. PHP was used to develop the dynamic web pages for user interface. The bespoke database was developed internally by the LSHTM Clinical Trials Unit. In the WOMAN-2 trial, data are collected at each participating site and transmitted directly to the Clinical Trials Unit via the database. If there is poor Internet connection, paper case report forms (CRFs) can be sent via email. Data checks and cleaning are performed by the Clinical Trials Unit. Data items to be coded including Adverse Event term and terms used to describe 'other' causes of death on the Outcome Form are coded using the Medical Dictionary for Regulatory Activities (MedDRA) Version 22.0 ^34^. The final database lock will take place at the end of the trial within three months from the time when the 'Last patient' in the 'Last follow-up' has completed the trial. Data will be exported for statistical analysis using the most recent version of Stata ^35^ or R ^36^.

### Data sharing

Following publication of the primary and secondary analyses detailed in this statistical analysis plan, the trial data will be made available via our data sharing portal: The Free Bank of Injury and emergency Research Data (freeBIRD) website ( http://freebird.Lshtm.ac.uk). This will allow for maximum use of the data to improve patient care and advance medical knowledge.

## Discussion

This statistical analysis plan is an update to our previously published protocol. The main change is an increase in the sample size from 10,000 to 15,000 patients. We present our plan for the statistical analyses in advance of the database lock and unblinding in order to guard against post hoc data dependent analyses. The WOMAN-2 trial should provide reliable evidence on the effect of tranexamic acid on PPH in women with moderate or severe anaemia.

## List of abbreviations

ANCOVA         Analysis of covariance

CI                     Confidence interval

CONSORT      Consolidated Standards of Reporting Trials

CRF                 Case report form

DMC                Data monitoring committee

DMP                Data management plan

DVT                 Deep vein thrombosis

GMP                Good manufacturing practice

MI                    Myocardial infarction

PE                    Pulmonary embolism

PPH                 Postpartum haemorrhage

RBC                Red blood cells

SAP                Statistical analysis plan

SIRS               Systemic inflammatory response syndrome

TSC                Trial steering committee

TXA               Tranexamic acid

VOE               Vascular occlusive event

WHO              World Health Organization

## Data Availability

No data are associated with this article.
