# Supplementary material for: Tranexamic acid for the prevention of postpartum bleeding in women with anaemia:  Statistical analysis plan for the WOMAN-2 trial: an international, randomised, placebo-controlled trial
Source: Gates Open Res. 2023 Aug 3;7:69. Originally published 2023 May 12. [Version 2] doi: 10.12688/gatesopenres.14529.2 (PMC10471795; doi:10.12688/gatesopenres.14529.2)
Supplement: Peer review history

# Peer review: Reviewer response for version 2

**DOI:** 10.21956/gatesopenres.16220.r34591

**Published:** 2023-08-31

**Authors:** Ferdousi Begum

**Affiliations:** 1 Ibrahim Medical College, Dhaka, Bangladesh

I think the revisions are appropriate.

Is the study design appropriate for the research question?

Partly

Is the rationale for, and objectives of, the study clearly described?

Yes

Are sufficient details of the methods provided to allow replication by others?

Partly

Are the datasets clearly presented in a useable and accessible format?

Partly

Reviewer Expertise:

NA

I confirm that I have read this submission and believe that I have an appropriate level of expertise to confirm that it is of an acceptable scientific standard.

# Peer review: Reviewer response for version 1

**DOI:** 10.21956/gatesopenres.15843.r33272

**Published:** 2023-07-10

**Authors:** Hadiza Galadanci

**Affiliations:** 1 Africa Center of Excellence for Population Health and Policy, Bayero University Kano, Kano, Nigeria

Thank you for inviting me to review the manuscript on “Tranexamic acid for the prevention of postpartum bleeding in women with anaemia: Statistical analysis plan for the WOMAN-2 trial: an international, randomised, placebo controlled trial”. It was very interesting to read and makes the reader eager to get the trial results. The manuscript is describing the analysis plan of WOMAN 2 Trial which is looking at the effect of tranexamic acid in preventing PPH in women with anaemia after vaginal delivery. Below are few of my comments.

1. Under “Trial and study Patients”, I feel it is important for the reader to be informed on what the eligibility criteria is for the study patients, even though a reference has been given for the WOMAN 2 trial protocol. When is the Haemoglobin test done to categorize the patient to moderate and severe anaemia and what are the values used. A summary of the criteria will enable the reader follow the manuscript without having to go back to the protocol.
2. Under “primary outcome” which is the amount of blood loss after delivery seems to be based on visual estimate of EBL, which as you know is very subjective and up to 53% can be missed as PPH (ref https://www.nejm.org/doi/full/10.1056/NEJMoa2303966 ^1^). I think it will be important to mention this as a limitation.
3. Under “other secondary outcome”, which quality of life questionnaire was used and was it validated for the population it was used for?

Is the study design appropriate for the research question?

Yes

Is the rationale for, and objectives of, the study clearly described?

Yes

Are sufficient details of the methods provided to allow replication by others?

Yes

Are the datasets clearly presented in a useable and accessible format?

Yes

Reviewer Expertise:

Feto maternal, Health Policy, Maternal Newborn Health

I confirm that I have read this submission and believe that I have an appropriate level of expertise to confirm that it is of an acceptable scientific standard.

## References

[1] : Randomized Trial of Early Detection and Treatment of Postpartum Hemorrhage. *N Engl J Med* .2023; 10.1056/NEJMoa2303966 10.1056/NEJMoa2303966 37158447

# Peer review: Reviewer response for version 1

**DOI:** 10.21956/gatesopenres.15843.r33901

**Published:** 2023-07-10

**Authors:** Ferdousi Begum

**Affiliations:** 1 Ibrahim Medical College, Dhaka, Bangladesh

Regarding the design of the Trial I have few questions for clear understanding and ethical issues regarding WOMAN-2 trial.

- The study design shows that women are randomized into intervention with TXA and control group. There is no mention whether AMTSL will be done or not for all patient of both the groups? Hope it will be done for all, which is a proven method to reduce PPH and PP blood loss.
- As in anemic pregnant mothers TXA (especially delayed or repeated use) may modify the coagulation factors, is there any effort to test coagulation factors of the patient? Will there be subgroup analysis plan?
- Are we seeing the effect of some factors e.g. Instrumental delivery; there is increased chance blood loss/PPH. Will there be subgroup analysis?
- How they are going to measure the blood loss during or after delivery till 24 hours. The blood collector can be used during delivery in dorsal / lithotomy position. But how the blood loss is measured when patient is lying down in bed/sitting/ standing.
- What is the end point/sign of diagnosis of PPH in respect to vital signs (lower limit Resp Rate, pulse rate, systolic / diastolic Blood Pressure).
- Are they measuring BMI- this may increase the incidence of PPH. Is there any variable on multiple Birth/polyhydramnios / placenta previa which may increase chance of PPH. Subgroup analyses may be done on separately for patient’s parity (multi /primi) also.
- Similar effect may happen due to previous history of C/S and TOLAC (Trial of Labor) VBAC (Vaginal Birth After Caesarean Section).
- What about the patients which Gestational /Chronic Diabetes Mellitus and /or Pregnancy Induced Hypertension all increases chance of induction of labor / difficult labor followed by PPH.
- Bleeding from big episiotomy / vulva hematoma may contribute to PPH.
- A large number of patients will be included in the study there are chance of obstructed labor, ruptured uterus and broad ligament hematoma. How these factors will be controlled/ analyzed?
- Pain relief was mentioned, but what kind of pain relief during labor will be used needs to be mentioned. It is very difficult to have anestheologist support for epidural anesthesia during labor pain in developing world where this study will be conducted.
- What kind of anesthesia will be used and how to the effects (use/ no use) will be analyzed?
- Is / will there be any Data Safety Monitoring Board (DSMB) formed? Because some patient will obviously die. We have to justify the effect of use of TXA VS no-TXA as cause of PPH/death.

Is the study design appropriate for the research question?

Partly

Is the rationale for, and objectives of, the study clearly described?

Yes

Are sufficient details of the methods provided to allow replication by others?

Partly

Are the datasets clearly presented in a useable and accessible format?

Partly

Reviewer Expertise:

NA

I confirm that I have read this submission and believe that I have an appropriate level of expertise to confirm that it is of an acceptable scientific standard, however I have significant reservations, as outlined above.
